# Continuous morphine infusion for end-stage lung cancer patients

**DOI:** 10.3892/ol.2012.1101

**Published:** 2012-12-28

**Authors:** YOUNG HAK KIM, CHIYUKI OKUDA, YUICHI SAKAMORI, KATSUHIRO MASAGO, YOSUKE TOGASHI, MICHIAKI MISHIMA

**Affiliations:** Department of Respiratory Medicine, Graduate School of Medicine, Kyoto University, Sakyo-ku, Kyoto 606-8507, Japan

**Keywords:** lung cancer, end-stage, morphine, intravenous, subcutaneous, pain, dyspnea

## Abstract

End-stage cancer patients frequently receive continuous morphine infusion (CMI) to alleviate the various symptoms associated with cancer progression or adverse events; however, there have been a limited number of studies concerning such patients. We conducted a retrospective analysis of 79 end-stage lung cancer patients who received CMI at the Kyoto University Hospital, Kyoto, Japan between 2008 and 2010. Thirty-one patients (39%) received CMI intravenously and 48 (61%) received it subcutaneously. The patients were divided into four groups based on the indications for CMI: group A (uncontrolled pain; n=9), group B (dyspnea; n=44), group C (both dyspnea and pain; n=13) and group D (an inability to take oral medicine; n=13). The median maximum dose of morphine in groups A-D was 60.0, 25.0, 50.0 and 15.0 mg/day, respectively. The median survival time from the start of CMI was 4 days (range 0–136). In our limited experience, pain, dyspnea and the inability to take oral medicine were identified as indications for CMI in end-stage lung cancer patients, with dyspnea being the major indication for CMI. Patients in group B (dyspnea) required a lower dose of morphine for alleviation compared with those in groups A (uncontrolled pain) and C (both dyspnea and pain). The survival time from the initiation of CMI was markedly shorter in patients with dyspnea (groups B and C) than in patients without dyspnea (group A). Further studies are required to facilitate the effective and appropriate use of CMI in end-stage lung cancer patients. Dyspnea was the major indication for CMI in end-stage lung cancer patients, and the survival time was extensively limited in such patients.

## Introduction

Cancer patients suffer from various symptoms, particularly in their final stage of life. Opioids play the main role in palliative care medicine, among which morphine is most commonly used (1). Although oral administration of morphine is preferable, a parenteral route is often used in terminally ill patients. Due to the inconvenience of intramuscular injections, either intravenous or subcutaneous injections are used. Continuous infusion limits the peak concentration, which reduces the adverse effects of morphine, such as sedation, and lessens the trough concentration problems of breakthrough pain or dyspnea. In addition, the dosage is easily titrated (2).

Lung cancer is the leading cause of cancer-related mortality, and its incidence is increasing worldwide. Lung cancer, along with lung cancer treatment and comorbid conditions, may cause various symptoms that require palliation. There have been retrospective analyses and reviews regarding continuous morphine infusion (CMI) for cancer patients (2,3); however, no such analysis limited to end-stage lung cancer patients has been reported.

In the present study, we conducted a retrospective analysis of end-stage lung cancer patients who received CMI at our hospital based on the indication for CMI.

## Patients and methods

A total of 79 patients with end-stage lung cancer who has been admitted to Kyoto University Hospital, Kyoto, Japan between 2008 and 2010 received CMI. Firstly, patient characteristics, including histology, initial treatment patients received, and infusion route, were analyzed. Then, the patients were divided by the major indications for CMI, and their clinical characteristics were compared between the groups. Survival time was measured from the start of CMI to the time of mortality. All patient data were obtained from our database. Informed consent approved by the Institutional Review Board was obtained from all patients.

## Results

### Patient characteristics

The patient characteristics are listed in Table I. The median patient age was 67 years (range, 34–86), and there were 55 males (70%) and 24 females (30%) in total. A total of 63 patients (80%) had been diagnosed with non-small-cell lung cancer (NSCLC) and 16 (20%) with SCLC. Sixty-five patients (82%) had undergone aggressive treatment and the remainder had received best supportive care. Forty-eight patients (61%) had received pre-infusion opioid administration, which comprised oxycodone, morphine sulfate and a fentanyl patch for 26, 11 and 11 patients, respectively. A total of 31 patients (39%) had received CMI intravenously and 48 (61%) received the treatment subcutaneously.

### Indications for CMI

All patients were divided into four groups based on the indications for CMI: Group A (uncontrolled pain; n=9), group B (dyspnea; n=44), group C (both dyspnea and pain; n=13) and group D (an inability to take oral medicine; n=13). The majority of the pain experienced was associated with cancer spread, and bone metastasis was most frequently observed (n=22). Dyspnea was cancer-related in a number of cases (carcinomatous pleurisy, n=22; airway constriction, n=8; carcinomatous lymphangiosis, n=6); however, in certain cases it was associated with treatment or comorbidity, such as interstitial pneumonia (n=12) and radiation pneumonitis (n=1). The major reason for the inability to take morphine orally was a decreased level of consciousness due to carcinomatous meningitis (n=8).

### Subgroup analysis based on the indications for CMI

**Figure 1:**
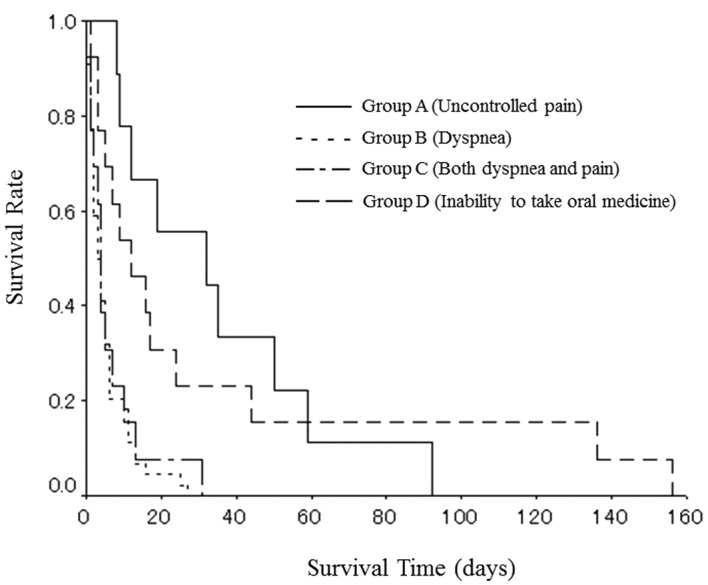
Survival curves of end-stage lung cancer patients who received continuous morphine infusion. The median survival time was 32 days in group A (uncontrolled pain), 3 days in group B (dyspnea), 4 days in group C (both dyspnea and pain) and 12 days in group D (inability to take oral medicine), respectively.

Table II summarizes the results of the subgroup analysis based on the indications for CMI. All patients with pain (groups A and C) had received pre-infusion opioids; however, two-thirds of patients without pain (group B) had not. With the exception of group D, both the initial and maximum doses were the lowest in group B, implying that dyspnea itself required a lower dose of morphine for alleviation compared with pain. The median survival time from the start of CMI was 32 days in group A, 3 days in group B, 4 days in group C and 12 days in group D (Fig. 1).

## Discussion

To the best of our knowledge, this is the first study of lung cancer patients who had received CMI in the final stage of their lives. In this analysis, uncontrolled pain, dyspnea and an inability to take oral medicine were identified as indications for CMI, and the patients were divided into four groups on this basis: Group A (uncontrolled pain), group B (dyspnea), group C (both dyspnea and pain) and group D (an inability to take oral medicine). Among the four groups, group D was an exception; patients in group D required CMI, not due to severe symptoms, but due to their inability to take oral medicine, mainly as a result of carcinomatous meningitis. Therefore, the degree of symptoms (pain or dyspnea) were observed to be markedly milder in group D compared with the other groups. With the exception of group D, dyspnea itself required a lower dose of morphine for alleviation, whereas the survival time from the start of CMI was shorter in patients with dyspnea (group B or C) than those without dyspnea (group A).

The prevalence of dyspnea varies with the primary tumor site. Dyspnea is one of the most commonly reported symptoms in lung cancer, with 15% of patients presenting with dyspnea at diagnosis and 65% suffering from it at some point during their illness. Close to death, 90% of patients with NSCLC suffer from dyspnea (4). In the present analysis, ∼70% of patients received CMI due to dyspnea (groups B and C). The efficacy of systemic morphine administration, both orally and parenterally, has been established in cancer patients with dyspnea (5,6).

In this study, 61% of patents received CMI intravenously and 39% received the treatment subcutaneously. The method of administration employed was selected by the doctor. In a prospective, within-patient, crossover study, continuous intravenous and subcutaneous morphine were demonstrated to be equally effective with a similar adverse effect profile (7). The parenteral route is desirable, either intravenously or subcutaneously, considering the respective advantages and disadvantages (8).

In conclusion, in our limited experience, terminally ill lung cancer patients required CMI due to uncontrolled pain, dyspnea and an inability to take oral medicine, and dyspnea was the major indication for CMI. Dyspnea required a lower dose of morphine for alleviation than uncontrolled pain; however, the survival time of patients who required CMI due to dyspnea was extremely short compared with patients without dyspnea. There has been no established strategy with regard to when to commence CMI and how to escalate the morphine dose. We predict that the strategy is likely to differ according to the indications for CMI, considering the apparent differences in the required morphine dose and the survival time between the patient subgroups in the present study. Further studies are required to facilitate the effective and appropriate use of CMI in end-stage lung cancer patients.

## Figures and Tables

**Table I:** Characteristics of the 79 end-stage lung cancer patients.

Characteristics	Value
Age (years)	
Median (range)	67 (34–86)
Gender, n (%)	
Male	55 (70)
Female	24 (30)
Histology, n (%)	
NSCLC	63 (80)
SCLC	16 (20)
Initial treatment, n (%)	
Chemotherapy	57 (72)
Chemoradiotherapy	6 (8)
Surgical resection	1 (1)
Radiation	1 (1)
Best supportive care	14 (18)
Pre-infusion opioids, n (%)	
Oxycodone	26 (33)
Morphine sulfate	11 (14)
Fentanyl patch	11 (14)
None	31 (39)
Infusion route, n (%)	
Intravenous	31 (39)
Subcutaneous	48 (61)

NSCLC, non-small-cell lung cancer; SCLC, small-cell lung cancer.

**Table II:** Subgroup analysis based on the indications for continuous morphine infusion.

Characteristic	Group A (n=9)	Group B (n=44)	Group C (n=13)	Group D (n=13)
Age (years), median (range)	72 (34–80)	48 (41–86)	61 (39–48)	66 (59–71)
Gender, male/female	6/3	27/17	12/1	10/3
Pre-infusion opioids, +/−	9/0	15/29	13/0	11/2
Infusion route, IV/SC	3/6	21/23	4/9	3/10
Starting-dose (mg/day), median (range)	50.0 (12.0–80.0)	25.0 (4.0–100.0)	25.0 (10.0–75.0)	10.0 (5.0–60.0)
Maximum-dose (mg/day), median (range)	60.0 (20.0–250.0)	25.0 (10.0–200.0)	50.0 (15.0–240.0)	15.0 (10.0–60.0)
Sedation, +/−	2/7	4/40	6/7	0/13

IV, intravenous; SC, subcutaneous.
